# Polycystic Ovary Syndrome: Etiology, Current Management, and Future Therapeutics

**DOI:** 10.3390/jcm12041454

**Published:** 2023-02-11

**Authors:** Samradhi Singh, Namrata Pal, Swasti Shubham, Devojit Kumar Sarma, Vinod Verma, Francesco Marotta, Manoj Kumar

**Affiliations:** 1 ICMR—National Institute for Research in Environmental Health, Bhopal Bypass Road, Bhopal 462030, India; 2 Stem Cell Research Centre, Department of Hematology, Sanjay Gandhi Post-Graduate Institute of MedicalSciences, Lucknow 226014, India; 3 ReGenera R&D International for Aging Intervention, 20144 Milano, Lombardia, Italy

**Keywords:** PCOS, gut microbiome, probiotics, FMT, gut dysbiosis, hyperinsulinemia, hyperandrogenism, metabolic disorders, miRNA therapy

## Abstract

Polycystic ovary syndrome (PCOS) is a complex endocrine and metabolic disorder, typically characterized by anovulation, infertility, obesity, insulin resistance, and polycystic ovaries. Lifestyle or diet, environmental pollutants, genetics, gut dysbiosis, neuroendocrine alterations, and obesity are among the risk factors that predispose females to PCOS. These factors might contribute to upsurging metabolic syndrome by causing hyperinsulinemia, oxidative stress, hyperandrogenism, impaired folliculogenesis, and irregular menstrual cycles. Dysbiosis of gut microbiota may play a pathogenic role in the development of PCOS. The restoration of gut microbiota by probiotics, prebiotics, or a fecal microbiota transplant (FMT) might serve as an innovative, efficient, and noninvasive way to prevent and mitigate PCOS. This review deliberates on the variety of risk factors potentially involved in the etiology, prevalence, and modulation of PCOS, in addition to plausible therapeutic interventions, including miRNA therapy and the eubiosis of gut microbiota, that may help treat and manage PCOS.

## 1. Introduction

One of the most prevalent endocrine system conditions affecting women of reproductive age is polycystic ovary syndrome (PCOS), also known as hyperandrogenic anovulation (HA) or Stein–Leventhal syndrome [1]. This chronic and heterogeneous disorder manifests itself as menstrual dysfunction, infertility, hirsutism, acne, and obesity [2]. It describes a condition where at least one ovary has an ovarian volume greater than 10 mL and at least one ovary has an estimated ten small cysts, with diameters ranging from 2 to 9 mm, develop [3]. It is usually only diagnosed when complications develop that significantly reduce a patient’s quality of life (e.g., hair loss, alopecia, acne, and infertility-related problems) [4]. According to a systematic screening of women using the National Institutes of Health (NIH) diagnostic standards, 4–10% of reproductive-age women are predicted to have PCOS worldwide [1]. The World Health Organization (WHO) estimates that in 2012 PCOS affected 116 million women (3.4%) globally [5]. This high frequency, as well as its link with ovulation and menstruation abnormalities, infertility, hair loss, and metabolic issues, underscores PCOS’s significant financial burden [2,6]. Although PCOS can occur at any age, beginning with menarche, the majority of instances are identified between the ages of 20 and 30 [7]. PCOS affects 1.55 million women of reproductive age worldwide, resulting in 0.43 million disability-adjusted life years (DALYs). The age-standardized incidence rate of PCOS in women of reproductive age was 82.44 per 100,000 in 2017, 1.45% higher than in 2007 [8]. Recent research reveals that PCOS is a lifelong syndrome that first manifests during pregnancy, although it was traditionally thought to be a disorder that only affected adult women [9]. While the exact cause of this multifactorial disorder is unknown, a combination of inherited and environmental factors is thought to play a primary role. The pathophysiology of PCOS is chiefly concerned with hormonal imbalance, chronic low-grade inflammation, insulin resistance, and hyperandrogenism, which impair folliculogenesis and increase the risk of related comorbidities, such as endometrial cancer and type II diabetes. According to international recommendations, the three main factors used to diagnose PCOS are hyperandrogenism, ovarian morphology, and anovulation [10]. A range of environmental factors, including geography, diet and nutrition, socioeconomic status, and environmental pollutants, are possibly contributing to the development, occurrence, and management of PCOS [11]. In recent years, the link between PCOS and the microbiome has been established, and it is believed to have contributed to the establishment of the syndrome. Dysbiosis of the gut microbial community, caused by environmental risk factors, might be a potential pathogenic factor in the development and progression of PCOS. Different pathogenic aspects of PCOS are caused by different microbiota, and essential routes linking their involvement in the onset of various clinical manifestations of PCOS bring up new therapy options for the condition [12]. Prebiotics, probiotics, synbiotics, and fecal microbiota transplants (FMTs) help manage the variety of phenotypes associated with PCOS by boosting eubiosis and reducing the impact of altered microbial profiles. Microbiota-mediated therapies might improve the metabolic, inflammatory, and hormonal characteristics of PCOS women.

This review summarizes the risk factors that may contribute to the development, prevalence, and modulation of PCOS, as well as its possible treatment approaches, including IL-22 and miRNA therapy. Additionally, we discuss the importance of gut dysbiosis in the pathogenesis of PCOS and evaluate several microbiota-focused intervention options that could help manage the disorder.

## 2. Phenotypes of PCOS

Four phenotypes have been identified by the medical community that can be regarded as variants of PCOS based on the three key parameters of PCOS, which are anovulation, hyperandrogenism, and polycystic ovaries (Table 1) [13]. The four phenotypes consistently range from the most severe (phenotype A) to the least severe (phenotype D) along an axis of metabolic and ovarian dysfunction [1].

## 3. Disease Pathophysiology

**Figure 1:**
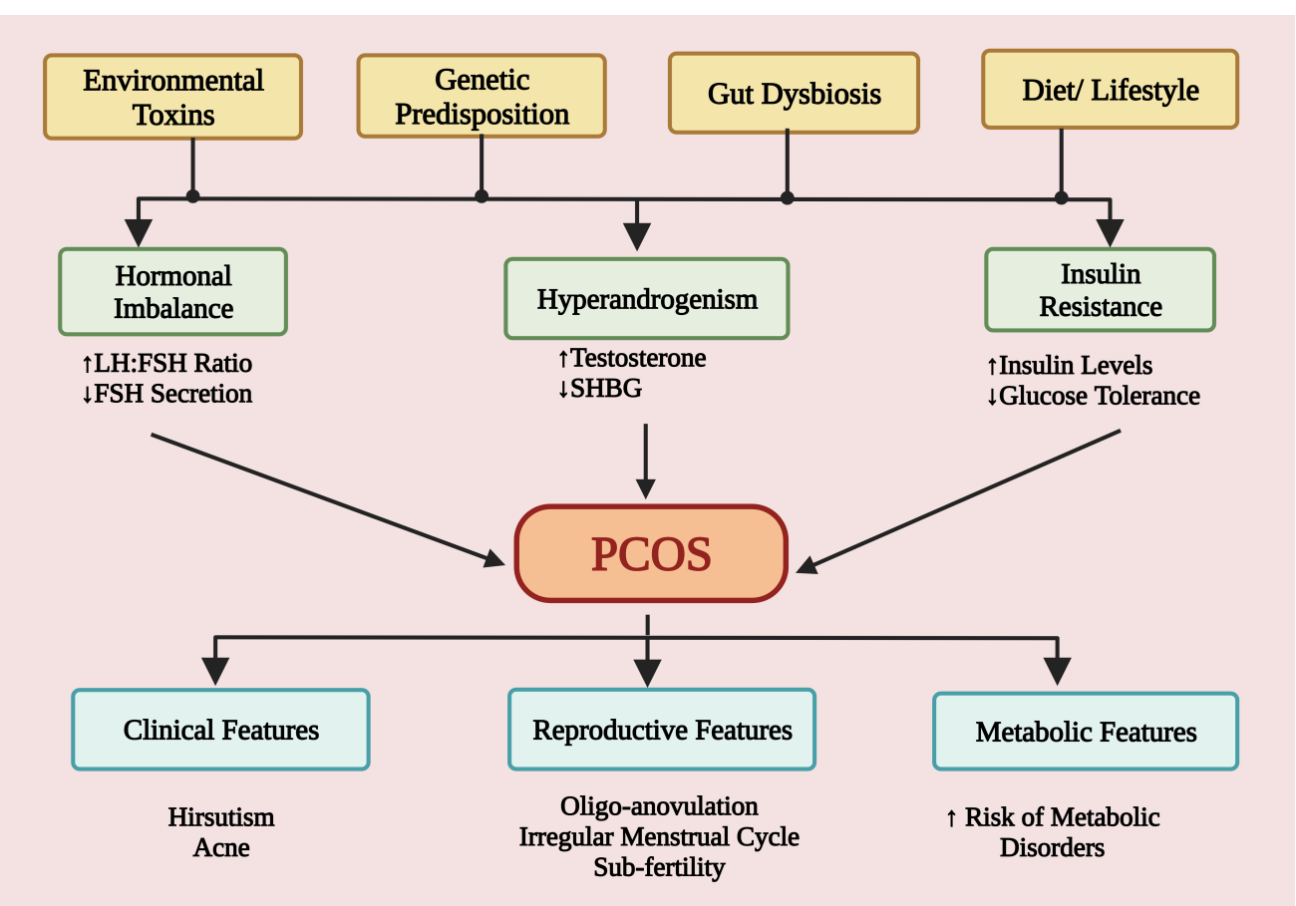
This schematic illustration shows the proposed pathophysiology and features of PCOS. The risk factors like environmental toxins, genetics, gut dysbiosis, and diet responsible for the pathophysiology of the PCOS and subsequent development of clinical, reproductive and metabolic features in PCOS patients. LH: luteinizing hormone; FSH: Follicle stimulating hormone; SHBG: Sex hormone binding globulin.

Across the globe, PCOS affects between 8% and 20% of women of reproductive age annually, according to the diagnostic criteria [14].The pathophysiology of this condition is influenced by alterations in steroidogenesis, ovarian folliculogenesis, neuroendocrine function, metabolism, insulin production, insulin sensitivity, adipose cell activity, inflammatory factors, and sympathetic nerve function [15]. According to Barre et al., the high consumption of carbohydrates, hyperinsulinemia, hyperandrogenemia, and persistent low-grade inflammation are the four key contributors to pathophysiological alterations in PCOS [16]. (Figure 1).

### 3.1. Hyperandrogenism

The biochemical hallmark of PCOS is hyperandrogenemia, which manifests clinically as hirsutism, acne, and alopecia. High levels of androgens are observed in 75–90% of PCOS patients with oligomenorrhea, and their concentrations frequently increase with the severity of the phenotype. Excessive androgen synthesis by the ovaries as well as the adrenals contributes to hyperandrogenism [17]. Increased levels of free (unbound) testosterone, a major hormone contributing to the pathogenesis of PCOS, are indicative of hyperandrogenism. Abnormal ovarian or adrenal function leads to the overproduction of androgens. In PCOS, impaired folliculogenesis is the initial effect of excess androgens disrupting normal androgen synthesis. At the early gonadotropin stage, excess androgens encourage the growth of primordial follicles and a rise in the antral follicles [18]. The release of gonadotropin hormones from the pituitary is triggered by GnRH production from the hypothalamus. To increase androgen synthesis in ovarian theca cells, luteinizing hormone (LH) activates the LH receptor. At the same time, follicle-stimulating hormone (FSH) activates the FSH receptor in ovarian granulosa cells to convert androgens into estrogens, which stimulate follicle growth. The dysregulation of the neuroendocrine system is thought to cause an imbalance in the hypothalamic–pituitary–ovarian (HPO) axis, which then leads to an excess of gonadotropin. The rise in GnRH promotes the production of LH over FSH, resulting in a substantial hormonal surge in the LH:FSH ratio in PCOS [19]. Theca cells in the ovaries undergo hyperplasia as a result of increased LH stimulation, which also causes a build-up of follicular fluid that forms cystic structures along the ovary’s periphery, giving it the appearance of a string of pearls. This is because many follicles in the theca cells of the ovaries become arrested, mostly in the preantral and antral stages. Due to a rise in follicles and the expression of essential enzymes involved in androgen synthesis, an excessive amount of androgens are produced [20].

An altered cortisol metabolism is another proposed mechanism that contributes to excess androgens in PCOS patients. The enhanced inactivation of cortisol by 5alpha-reductase (5alpha-R), or the impaired reactivation of cortisol from cortisone by 11 beta-hydroxysteroid dehydrogenase type 1 (11beta-HSD1), may cause increased peripheral cortisol metabolism, which results in less negative feedback suppression of adrenocorticotropic hormone (ACTH) secretion while maintaining normal plasma cortisol concentrations at the expense of excess androgens [21]. Various genetic factors are associated with abnormal steroidogenesis. CYP genes involved in steroidogenesis play an important role in androgen production and are considered key players in hyperandrogenism in PCOS [20].

### 3.2. Hyperinsulinemia

Insulin is the main hormone in charge of both lipogenesis and glucose homeostasis. Insulin serves as a mitogenic hormone in addition to having an impact on the metabolism of carbohydrates, fats, and proteins. Insulin receptors, which are present in many tissues of the HPO axis, mediate the activities of insulin. Insulin potentiates the corresponding trophic hormones in steroidogenic tissues, such as the ovary and the adrenal cortex, to encourage steroidogenesis [14]. As insulin directly mimics the action of LH and indirectly raises GnRH, hyperinsulinemia is the primary cause of excessive androgen production. Sex hormone binding globulin (SHBG), a key circulatory protein that regulates testosterone levels, is decreased by insulin. Therefore, lower SHBG levels would lead to higher levels of free androgens, which cause clinical symptoms of PCOS, such as hirsutism, alopecia, and acne [18]. Numerous studies have shown that lowering insulin resistance will ultimately result in reduced androgens and an improvement in the disease condition [22,23,24].

## 4. Causes and Risk Factors

It is difficult to identify the causative factors of this multifactorial condition due to its complex interlinked pathophysiology. The origin, prevalence, and modulation of the PCOS phenotype may be affected by environmental pollutants, diet and lifestyle choices, genetic factors, obesity, and gut dysbiosis. These factors might lead to the cause of excessive androgen secretion from the ovaries, the onset of insulin resistance, partial folliculogenesis arrest, and the chronic low-grade release of inflammatory mediators from white blood cells, upsurging metabolic syndrome.

### 4.1. Etiological Role of Environmental Pollutants

Various studies have shown that environmental pollutants, such as heavy metals, insecticides, and endocrine-disrupting chemicals (EDCs), significantly affect human health and reproduction. Indeed, there is mounting evidence that environmental pollutants contribute to the development of PCOS. Takeuchi and Kandaraki et al. discovered that serum BPA levels in hyperandrogenic women with PCOS were greater than in non-hyperandrogenic women with PCOS and healthy controls [25,26]. A separate study found that increases in blood BPA levels were positively associated with serum testosterone levels in PCOS women versus healthy women [27]. To ascertain the relationship between various environmental pollutants and PCOS, Vagi et al. carried out a case–control study that reported higher serum levels of perfluorooctanoate and perfluorooctane sulfonate in women with PCOS [28]. A negative association between phthalate body burden and PCOS was also noted by the group. To be more precise, mono benzyl phthalate (mBzP) urine concentrations were lower in PCOS-afflicted women [28], indicating an impaired xenobiotic metabolism. Previously, it was thought that EDCs, including BPA and phthalates, mostly affected thyroid, estrogen, progesterone, and androgen receptors in the nuclear hormone system; however, subsequent research has revealed that, in addition to other reproductive routes, EDCs can influence nonnuclear hormone receptors, orphan receptors, and receptors for neurotransmitters, as well as being able to directly change steroidogenesis and hormonal metabolism [29]. EDCs are a group of ubiquitous contaminants that have been well-researched as potential environmental contributors to the pathophysiology of PCOS. This disorder is linked to higher levels of oxidative stress and inflammation, which contribute to insulin resistance, obesity, and infertility; all these dysfunctions can be connected with EDC exposure, either directly or indirectly. Additionally, PCOS has neurotransmitter profile alterations similar to those seen in animals exposed to EDCs [30]. Endocrine disruptors are implicated in the induction of metabolic and reproductive abnormalities that resemble PCOS symptoms in vitro and animal investigations [31]. It is conceivable that developmental exposure to particular EDCs could alter metabolic, reproductive, and neuroendocrine regulation permanently in ways that favor PCOS development in people who are genetically predisposed to it, or that it could hasten and/or exacerbate the syndrome’s natural course throughout life cycle exposure [31]. EDCs may also result in epigenetic alterations in the DNA of the female reproductive system that may affect subsequent generations and result in the transmission of potential PCOS traits [32]. Overall, EDCs can interfere with the regulation of hypothalamic–gonadal hormones, as well as local paracrine and autocrine systems, which can eventually result in PCOS pathogenesis.

Numerous studies have found a positive correlation between PCOS incidence, smoking, and exposure to cigarette smoke [33,34,35]. Smoking was found to be associated with ovulatory dysfunction in a dose-dependent manner in a study that included oligo-anovulatory women with PCOS, women with normal anovulation in PCOS, and healthy controls [33]. An inflammatory condition characterized by an increase in mononuclear cells, mitochondrial dysfunction, a reduction in GSH (glutathione) as well as oxygen uptake, and an oxidative state with decreased antioxidant levels are all well-associated with PCOS and smoking [34]. These inflammatory stimuli can, in turn, alter the enzymes and cause steroidogenesis in theca cells. Polycyclic aromatic hydrocarbons (PAHs) produced from cigarette smoke, burnt coal, gas, wood, garbage, and meat cooked at high temperatures constitute a major part of the air pollutants that are positively correlated with the risk of developing PCOS [35]. Air pollutants, such as nitrogen oxides, sulfur dioxide, PAHs, and particulate matter (PM) 2.5, may increase inflammatory mediators in exposed women and alter normal steroidogenesis, which may contribute to the development of PCOS. Results from a population-based cohort study conducted in Taiwan showed that increased exposure to fine air pollutant particles and pollutant gases, namely SO_2_, NO, NO_2_, NOx, and PM_2.5_, was associated with increased PCOS risk [36]. A connection between PCOS and environmental pollutants is also supported by animal models. According to recent research, the direct exposure of pregnant rats to either fungicide vinclozolin or insecticide DDT was associated with the development of ovarian abnormalities consistent with PCOS in three subsequent generations via epigenetic processes [37,38].

### 4.2. Role of Diet and Lifestyle

Lifestyle changes are the primary line of treatment for women with PCOS, but they are not an alternative to pharmacological treatments. Regular physical exercise, maintaining a healthy body weight, adhering to healthy dietary habits, and abstaining from tobacco use are all important in the prevention and treatment of metabolic diseases, and are recommended in clinical guidelines for a variety of ailments.

High-calorie diets and sedentary lifestyles might be possible causes of exacerbating PCOS. High-sugar diets may contribute to PCOS by altering gut flora, inducing chronic inflammation, increasing insulin resistance, and increasing androgen production. Obesity and weight gain worsen defining features of this syndrome. In comparison to high-glycemic-index (HGI) meals, low-GI (LGI) diets reduced fasting insulin, total and LDL-C, TGs, waist circumference, and total testosterone without altering fasting glucose, HDL-C, weight, or the free androgen index in PCOS patients. Additionally, the addition of an LGI diet, exercise, and/or omega-3 supplementation boosted HDL, SHBG synthesis, and body fat reduction [39,40]. Gonzales et al. reported that saturated fat ingestion promotes LPS-mediated inflammation and insulin resistance by enhancing the circulation of TNF-α and peripheral leukocytic suppressor of cytokine-3 (SOCS-3) expression in PCOS [41]. As a result, removing saturated fats from these patients’ diets is critical. In rats, dietary α-linolenic-acid-rich flaxseed oil ameliorated PCOS via the sex steroid hormone–microbiota–inflammation axis, although other sources of α-linolenic acid are likely to generate an equivalent effect [42]. Based on epidemiological evidence and, more recently, confirmed via genetic investigations, obesity and PCOS are closely related [43]. PCOS is aggravated by obesity, primarily through worsening insulin resistance (IR). Obesity has been linked to PCOS development due to aberrant activity of the HPO axis. Obesity is associated with hyperinsulinemia, which worsens PCOS patients’ glucose intolerance and lipid profile. By stimulating LH, obesity increases androgen production, which in turn causes hyperandrogenism [44]. Fermentable fiber offers metabolic benefits for the gut flora, resulting in the release of SCFAs [45]. Low-GI diets may alter appetite-regulating hormones, such as ghrelin and glucagon [46,47]. In women with PCOS, low-GI meals lowered ghrelin while increasing glucagon [47]. High fructose consumption (HFC) exacerbated endocrine but not metabolic alterations in PCOS, implying that HFC may worsen endocrine-related phenotypes in PCOS [48]. A meta-analysis and systematic review concluded that an LGI diet is an effective, acceptable, and safe intervention for IR relief, and that professional dietary counselling should be provided to all PCOS patients [49,50]. Another low-GI diet adjustment appears to be a ketogenic diet (KD), which restricts total carbohydrate consumption in favor of plant-based fat. In obese women with PCOS and liver dysfunction, a KD improves the menstrual cycle, lowers blood glucose and body weight, improves liver function, and treats fatty liver [51]. Paoli et al. discovered even more intriguing results after utilizing KDs for 12 weeks in women with PCOS [52]. Anthropometric and body composition measures demonstrated a considerable decrease in body weight, BMI, and fat-free body mass. There was a considerable decrease in glucose and insulin blood levels, as well as a significant improvement in HOMA-IR scores. TGs, total cholesterol, and LDL levels all decreased significantly, while HDL levels increased. In addition, estradiol, progesterone, and SHBG levels increased, while the LH/FSH ratio, LH total as well as free testosterone, and DHEAS blood levels decreased dramatically [52]. Therefore, a KD may yield even better benefits than a diet with an LGI in PCOS patients who have severe obesity and/or obesity coupled with full-blown metabolic syndrome; however, a general conclusion may be drawn that, by adhering to the fundamentals of a healthy diet, physiological homeostasis can be regulated and faster disease recovery can be accomplished. Women with PCOS who lose excess weight through alterations in their lifestyles experience regulated menstruation and improved reproductive outcomes [53]. Physical exercise in the management of PCOS is becoming more recognized and accepted among patients as well as professionals in the health sector [54]. By optimizing glucose transport and metabolism, physical exercise enhances the effects of insulin sensitivity. Improvements in health outcomes are more reliant on exercise intensity than dose, according to a recent meta-analysis. The analyses’ findings show that exercise is beneficial and suggest that vigorous exercise may have the biggest effects on body composition, insulin resistance, and cardiorespiratory fitness [55]. Women with PCOS should engage in vigorous aerobic exercise and resistance training to improve their insulin sensitivity and androgen levels [54].

### 4.3. Role of Genes and Genetics

**Figure 2:**
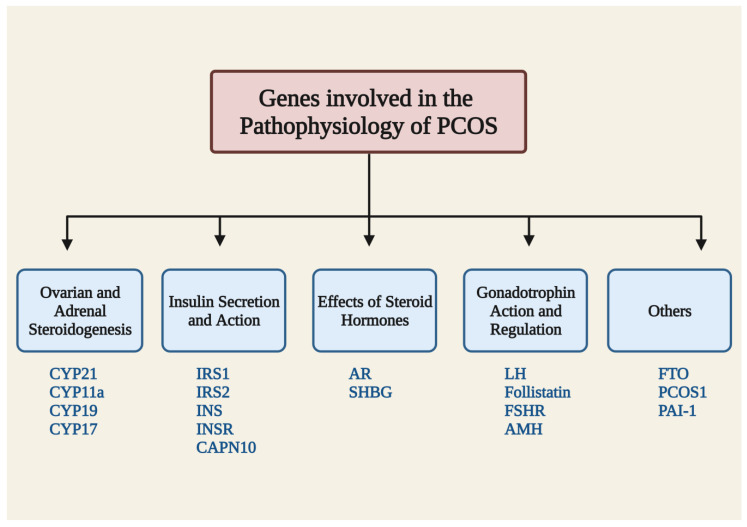
Summary of the genes involved in the pathophysiology of PCOS. CYP: cytochrome family p450; IRS: insulin receptor substrate; INS: insulin gene; INSR: insulin receptor; AR: androgen receptor gene; SHBG: sex hormone binding globulin; FSHR: follicle-stimulating hormone receptor; LH: lutein hormone; AMH: anti-Mullerian hormone; FTO: fat mass obesity; PAI-1: plasminogen activator inhibitor 1; and CAPN10: caplain-10.

PCOS is a polygenic and multifaceted disorder, and it has been shown that certain genes, gene–gene interactions, or interactions between genes and the environment might affect a person’s propensity to develop PCOS [56]. Several genetic studies have revealed that several potential genes with single-nucleotide polymorphisms or mutations are connected to a variety of PCOS symptoms. PCOS is linked to all genes and mutations that directly or indirectly impact the ovaries [57]. The pathophysiology of PCOS is most frequently mediated by genes encoding signaling elements related to steroidogenesis, steroid hormone action, gonadotrophin action and control, insulin action and secretion, energy metabolism, and chronic inflammation (Figure 2) [57,58]. Finding significant gene variants that may alter a gene’s expression or subsequent protein function is necessary to delineate the genetic architecture of this complex disorder. The identification of genetic markers may enhance the diagnosis of this syndrome, enabling early intervention in co-morbidities that are related to the syndrome as well as its phenotypes and more tailored treatments.

### 4.4. Gut Microbiota Dysbiosis: Critical Correlation

The gut microbiome is made up of approximately 10^13^ to 10^14^ microorganisms that collectively have almost 200 times more genes than the human genome, making it an “organ” on its own [59]. Gut dysbiosis appears to be the root of the inflammation and alteration of gut permeability, which can then affect a host’s health. Under physiological conditions, a delicate balance exists between the gut microbiota and the host that influences physiology, metabolism, nutrition, and immune function, in addition to playing a significant role in the prevention of various diseases. Between healthy adults there are substantial variances in microbiome composition, and these variations may contribute to susceptibility to certain diseases.

Numerous studies in recent years have examined the connection between PCOS and alterations in the gut microbiota [60,61,62]. These investigations revealed a significant difference in the composition of the gut microbiome between healthy controls and PCOS patients. Studies indicate that the diversity and structure of the gut microbiota in PCOS-affected women may be impacted by insulin resistance, sex hormone levels, and obesity [53]. Gut microbiota and their metabolites share a close association with PCOS. Significant differences between PCOS and the control group were found in the number of species and metabolites produced, mostly indicated by a decline in microbial diversity, which was characterized by a decrease in beneficial bacteria (Lactobacilli and Bifidobacteria) and an increase in pathogenic bacteria (Escherichia and Shigella) [60,61]. Gut microbiota metabolize the substrates entering the gut via diets and produce metabolites that may act directly on the intestines or enter systemic circulation and influence various host tissues such as the ovary, liver, skeletal muscle, and adipose tissue, whose functions are altered in PCOS. Secondary bile acids, short-chain fatty acids (SCFAs), and trimethylamine (TMA) are a few gut bacterial metabolites altered in PCOS [62]. Numerous human and rodent model studies have shown an association between changes in gut microbiota and PCOS, including a decline in biodiversity and alterations in particular bacterial taxa, despite the wide variation in the results from 16S rRNA and metagenomic gene sequencing. Studies have reported a change in α and β diversity as well as an alteration in the balance of some species of bacteria, such as Bacteroidetes and Firmicutes, in patients with PCOS. Three basic aspects— of the syndrome anovulation/menstrual irregularity, hyperandrogenism (acne, hirsutism), and the emergence of numerous small ovarian cysts—can be explained by the dysbiosis of gut microbiota (DOGMA) theory of PCOS [63]. According to the DOGMA theory, poor-diet-induced gut microbiota dysbiosis may lead to an increase in the permeability of the gut mucosa, which in turn increases the passage of lipopolysaccarides (LPS) from Gram-negative colonic bacteria into the blood stream. The resulting immune system activity disrupts insulin receptor function, elevating serum insulin levels, increasing the production of androgens in the ovaries, and interfering with normal follicle formation [63].

## 5. Treatment and Management

Treatments of PCOS must be tailored to the specific needs of each patient; goals of therapy may include ameliorating hyperandrogenic symptoms, inducing ovulation, regulating menstruation, and preventing cardiometabolic complications. For women with PCOS, irregular menstruation, hirsutism, and infertility are the most distressing symptoms. Due to the complex etiology of PCOS, its treatment is rarely monotherapeutic, rather being personalized based on prevailing signs and symptoms. Several complementary therapies have been suggested for the management and treatment of PCOS. Diet and lifestyle changes are regarded as the cornerstone of PCOS management. Different pharmacological and non-pharmacological interventions can be used to relieve the most prominent symptoms of PCOS, such as menstrual irregularities, androgen-related symptoms, and infertility-causing anovulation. For the regulation of metabolic comorbidities in PCOS, there are numerous therapeutic approaches with potential benefits; however, it is also crucial to acknowledge that no single treatment can fully address the range of metabolic abnormalities in PCOS-diagnosed women. Combining lifestyle changes with medications for various ailments results in greater metabolic benefits and improvements in metabolic comorbidity parameters than monotherapies do. Additionally, treatment should also take into consideration increased levels of anti-Müllerian hormone (AMH), plasma metabolomics, and gut microbiota composition, which are severe characteristics of PCOS, in addition to focusing on primary traits.

### 5.1. Oral Contraceptives and Anti-Androgens

Oral contraceptives (OCs) are the first-line management protocol for menstrual abnormalities and hirsutism/acne in women with PCOS [64]. OCs function by encouraging negative feedback on LH secretion, which leads to less androgen production in the ovaries and reduces hyperandrogenism. They raise liver-produced SHBG while lowering blood levels of free androgens. OCs also work by inhibiting the peripheral conversion of testosterone into dihydrotestosterone(DHT), binding DHT to androgen receptors, and decreasing the release of adrenal androgens [65]. The risk–benefit ratios of OC preparations can vary depending on their doses and medication combinations. The majority of OC preparations include estrogen (ethinylestradiol) and anti-androgens, such as cyproterone acetate (CPA), drospirenone, norgestimate, levonorgestrel, and desogestrel [66]. Anti-androgens, including spironolactone, CPA, flutamide, and finasteride, systematically lower levels of androgens [66], therefore being used in the medical management of hyperandrogenism. Antiandrogens are frequently used to treat PCOS because they help with hirsutism and other androgen-related issues. The anti-androgens have slightly distinct mechanisms of action, but they all impede testosterone’s function. Anti-androgen receptor drugs have been effective in treating PCOS characteristics. The main result of using OCs is a decrease in hyperandrogenism due to its effects on the hypothalamus and pituitary, in addition to ovarian steroidogenesis [67]. Due to these effects it is an effective pharmacological intervention for the treatment of menstrual irregularity, acne, hirsutism, and androgenic alopecia linked to PCOS [68,69]. Third-generation combination OCs, which contain antiandrogenic compounds, have been shown to improve the metabolic phenotypes of PCOS, lipid, and adipokine profiles in patients. The most prevalent competitive antagonist of ARs, flutamide, has been shown to benefit PCOS-affected women by reducing hirsutism and acne [70,71,72]. Patients with PCOS receiving flutamide medication also reported improved ovulation and menstrual cycle regularity [73,74]. Additionally, therapy with flutamide in both obese and lean PCOS women showed that flutamide improved the lipid profiles of women with PCOS, with a substantial decrease in total cholesterol, LDL, and TGs, regardless of weight changes [75]. Steroidal AR blockers, such as CPA and spironolactone, compete with T and DHT for binding to ARs. In PCOS patients, both of these AR blockers have been found to dramatically reduce hirsutism and acne [76]. Additionally, spironolactone medication was found, in one trial, to improve metabolic characteristics in PCOS-affected individuals [77]. Finasteride, a 5-alpha reductase inhibitor that inhibits the conversion of T into DHT, is another treatment used to effectively manage hirsutism and alleviate hyperandrogenic symptoms in PCOS patients [78,79]. When considered collectively, findings from the use of anti-androgenic medications in PCOS patients, either alone or in combination, have shown that the targeted reduction in hyperandrogenism and consequently androgenic activity has a positive effect, with improvements seen in a variety of PCOS traits. Comprehensive screening should be performed on women with PCOS to identify risk factors for severe side effects from OCs, such as a history of smoking, the presence of hypertension and obesity, and a history of clotting issues, to name a few very important factors.

### 5.2. Insulin Sensitizers

Defective insulin secretion and function are part of the pathophysiology of PCOS [80]. The elevated levels of androgens in PCOS are known to be influenced by hyperinsulinemia and insulin resistance. Ovarian function is regulated by insulin, and excessive insulin levels can have negative effects on ovarian function. In reaction to excessive insulin, theca cells release large levels of androgens, which in turn cause follicular maturation to be arrested, which increases the risk of polycystic ovarian morphology, a sign of PCOS [80]. In addition to playing a crucial part in the pathophysiology of PCOS, insulin resistance negatively affects PCOS patients by predisposing them to long-term health issues, such as T2DM and CVD. To effectively manage PCOS, a therapeutic approach that addresses insulin resistance, including pharmaceutical and lifestyle changes, is essential. By reducing insulin secretion and stabilizing glucose tolerance, insulin sensitizers increase insulin sensitivity in target tissues [81]. It has been demonstrated that insulin sensitizers, such as metformin and thiazolidinediones (TZDs), can trigger ovulation by reducing insulin resistance. Metformin (a biguanide) use is linked to improved ovulation, decreased levels of circulating androgens, and enhanced menstrual cyclicity [66]. It acts by reducing hepatic glucose synthesis, increasing glucose absorption, and improving peripheral tissues’ sensitivity to insulin. In research comparing metformin and lifestyle interventions in PCOS-afflicted women both groups experienced a significant decrease in BMI; however, only the metformin group experienced a decrease in testosterone levels [82]. Another RCT evaluating the impact of metformin on body weight in obese and severely obese PCOS women found that the drug significantly reduced BMI without the need for lifestyle changes [83]. Metformin has been shown in numerous trials to have a significant effect on dyslipidemia [84,85]. It either directly affects the hepatic metabolism of free fatty acids or indirectly acts by lowering hyperinsulinemia to improve dyslipidemia [86]. Additionally, TZDs (pioglitazone and rosiglitazone) lower insulin levels by increasing insulin sensitivity, which reduces levels of androgens in the blood [87]. Women with PCOS have reported that pioglitazone had an impact on reducing insulin resistance, hyperandrogenism, and ovulatory dysfunction. It significantly decreased fasting serum insulin and free androgen levels while increasing SHBG levels in an RCT that compared the effectiveness of the drug vs. a placebo in PCOS patients [88]. In a meta-analysis comparing the effectiveness of metformin and pioglitazone in treating PCOS, the pioglitazone group showed a substantial improvement in ovulation and the menstrual cycle [89]. According to the findings of a meta-analysis of 22 trials for women with PCOS, metformin combined with TZD appear to be more effective than metformin alone in improving insulin resistance and lipid metabolism while lowering total testosterone levels [90].

### 5.3. Ovulation Inducers

Ovulatory dysfunction is one of the diagnostic criteria for PCOS patients, and ovulation induction is an effective treatment for PCOS patients with fertility requirements. Anovulation in PCOS is associated with low FSH levels and the arrest of antral follicle growth during its final stages of maturation. The overproduction of LH, androgens, and insulin may all work together or separately to influence this process directly or indirectly, enhancing steroidogenesis but preventing follicular growth. The first-line medication for ovulation induction is still clomiphene citrate (CC), a partially selective estrogen receptor modulator [91]. As an estrogen receptor antagonist, CC inhibits negative feedback in the estrogen signaling pathway, leading to increased FSH availability. Increased FSH causes follicular growth, which is followed by an LH surge and ovulation. Low-dose gonadotropin therapy can also be used for the induction of ovulation and mono-follicular development [92]. It is thought that women with PCOS have a relative decrease in aromatase, which reduces the production of follicles responsible for effective ovulation. Aromatase inhibitors (AIs) are considered to induce ovulation because of their selective action of blocking the conversion of androgens into estrogens in ovarian follicles, peripheral tissues, and the brain, creating a positive feedback loop with the estrogen of the HPO axis, which causes the endogenous release of GnRH, promotes FSH secretion, and causes follicular growth. Selective Ais, such as letrozole and anastrozole, have been suggested as primary and secondary treatments for ovulation induction [76,93,94]. Letrozole has the advantage of avoiding peripheral antiestrogenic effects on the endometrium while stimulating mono-follicular growth [95].

### 5.4. Calcium and Vitamin D Supplements

Vitamin D plays a physiologic role in reproduction, including in ovarian follicular development and luteinization, via altering AMH signaling, FSH sensitivity, and progesterone production in human granulosa cells. Through a variety of functions, it also impacts glucose homeostasis [96]. The presence of a specific vitamin D receptor (VDR) in pancreatic β-cells and skeletal muscle, the expression of the enzyme 1α-hydroxylase, which can catalyze the conversion of 25-hydroxyvitamin D [25(OH)D] into 1,25-dihydroxyvitamin D, and the presence of a vitamin D response element in the human insulin gene promoter are some of the potential effects of vitamin D on glucose homeostasis [96]. Low 25(OH)D levels may aggravate PCOS symptoms, such as insulin resistance, ovulatory and menstrual irregularities, infertility, hyperandrogenism, and obesity, as well as increase the risk of cardiovascular disease. In vitamin-D-deficient individuals with PCOS, vitamin D administration can lower abnormally increased serum AMH levels while increasing serum anti-inflammatory soluble receptors for advanced glycation end products. In particular, vitamin D and calcium supplementation, in addition to metformin therapy, may improve menstrual regularity, ovulation, hyperandrogenism, and follicular development in PCOS patients [97,98]. Women with PCOS have high AMH levels, which lead to aberrant ovarian folliculogenesis. Vitamin D therapy restores serum AMH levels, which may lead to improved folliculogenesis [99]. Results from a meta-analysis showed that combining metformin with a calcium/vitamin D supplement improved menstrual regularity and follicular maturation, significantly reduced serum insulin levels, fasting blood sugar (FBS), and homeostasis model assessment insulin resistance (HOMA-IR), and significantly increased the quantitative insulin sensitivity check index (QUICKI). Additionally, it decreased hirsutism and testosterone levels, serum TG and VLDL-C levels, and cholesterol as well as LDL levels in PCOS patients [100].

## 6. Emerging Therapeutics

### 6.1. Statins

Women with PCOS frequently have dyslipidemia, which is a significant predictor of cardiovascular risk due to increased LDL-C, triglyceride (TG), and low HDL-C levels [101]. Therefore, the effective treatment of PCOS involves improving the lipid profile and subsequently reducing cardiovascular disease morbidity. Statins (atorvastatin, pravastatin, rosuvastatin, fluvastatin, and simvastatin) help treat PCOS because they lower sex steroid production, improve dyslipidemia, improve inflammation, and lower ovarian androgen production by preventing thecal cells from producing androgen [102]. The rate-regulating enzyme 3-hydroxy-3-methylglutaryl coenzyme A (HMG-CoA) reductase, which is necessary for the process of cholesterol production, is inhibited by statins. By blocking this enzyme, cholesterol formation will also be prevented because HMG-CoA will not be converted into mevalonate [103]. When the atorvastatin and placebo groups were both given metformin for another 12 weeks, the atorvastatin pretreated group significantly outperformed the placebo pretreated group in terms of HOMA-IR, the free androgen index (FAI), total testosterone, and SHBG, indicating that atorvastatin enhances the effect of metformin [104]. In this investigation, atorvastatin dramatically decreased acylation stimulating protein (ASP), IL-6, and monocyte chemoattractant protein-1 (MCP-1), which are markers of inflammation and adipose tissue dysfunction, followed by a significant improvement in HOMA-IR and testosterone levels [105]. In randomized placebo-controlled research, atorvastatin significantly decreased hyperandrogenism, inflammatory markers, and insulin resistance in PCOS-affected women compared to a placebo [106,107]. A measure of oxidative stress, serum malondialdehyde (MDA), was dramatically lowered in obese women with PCOS receiving atorvastatin treatment [108]. In addition, the group of PCOS-afflicted women had considerably lower levels of androstenedione and dehydroepiandrosterone sulfate (DHEAS) after taking atorvastatin [109]. Additionally, compared to a placebo, a 12-week course of atorvastatin significantly increased the levels of serum vitamin D (25OH-D) in PCOS women [104]. The results of a meta-analysis of nine RCTs supported the use of statins as a viable treatment for PCOS by showing that they could lower androgen levels and improve the cutaneous symptoms of hyperandrogenism in PCOS patients [110]. Another meta-analysis revealed that the statin group experienced a significant drop in total testosterone, free testosterone, androstenedione, DHEAS, LH, the LH-to-FSH ratio, and prolactin. In addition to demonstrating a significant drop in fasting glucose, the insulin sensitivity index, and high-sensitivity C-reactive protein, the study also showed a significant decline in total cholesterol, LDL-C, and TGs in the statin group [111].

### 6.2. Glucagon-Like Peptide-1 (GLP-1) Agonist

Incretin hormones, such as glucagon-like peptide-1 (GLP-1) and glucose-dependent insulinotropic polypeptide (GIP), are well-known stimulators of glucose-dependent insulin release, particularly after a meal, a phenomenon known as the incretin effect [112]. In cases of insulin resistance, incretin action is frequently impaired. According to a recent study, lower levels of the incretin hormone have been found in PCOS patients. This being the case, targeting this mechanism might be a useful therapeutic strategy for PCOS treatment. Given that insulin resistance is the primary cause of metabolic and endocrine dysfunction in PCOS, GLP-1 agonist therapy has obvious therapeutic benefits in this population. Although the primary effect of GLP-1 agonists is not to stimulate insulin secretion, their weight loss effect may indirectly improve insulin sensitivity. According to a study, these agents improve insulin sensitivity by acting on eight different molecular pathways, including those of inflammation, oxidative stress, lipid metabolism, GLUT-4 expression/translation, β-cell function, the endoplasmic reticulum (ER), and insulin signaling [113]. Commercially available GLP-1 agonists include liraglutide, semaglutide, dulaglutide, and exenatide. A recent systematic review and meta-analysis that compared the efficacy of GLP-1 agonists and metformin in women with PCOS have shown a significant improvement in insulin sensitivity, reduced BMI, and abdominal girth compared with metformin [114]. In addition, GLP-1 receptor agonist therapy has shown positive results in terms of weight reduction and a decrease in testosterone levels in obese women with PCOS [115]. Another study that examined the effect of liraglutide on depression and quality of life (QOL) in obese PCOS patients found a significant improvement in QOL with dramatic weight loss [116]. Dual GLP-1/GIP receptor agonists (twincretins) have shown better potency in inducing glycemic control and weight loss, reducing hepatic fat content, and improving adiposity, lipid profiles, and metabolic parameters than GLP-1 agonists alone in different disease models. From the current data, it would seem that these are promising new therapies with potential utility for PCOS treatment. Therefore, these could potentially be novel therapeutic options in women with PCOS to improve metabolic risk if proven beneficial in clinical studies.

### 6.3. Inositols

Inositol is a carbocyclic sugar found in high concentrations in human and plant cells. It exists in nine different isomeric forms, the most common of which are myoinositol (MI) and d-chiro-inositol (DCI). The inositols found in fruits and beans are incorporated into cell membranes as phosphatidyl-MI, a precursor of inositol triphosphate (InsP3). InsP3 acts as an intracellular second messenger and regulates a variety of hormones, including thyroid-stimulating hormone (TSH), FSH, and insulin [117]. MI-based secondary messenger activation regulates glucose intake by increasing the activity of glucose transport proteins, whereas DCI secondary messenger activation stimulates glycogen synthesis. Defects in this pathway can cause insulin resistance by impairing insulin signaling. The enzyme epimerase converts MI into DCI while maintaining a physiological ratio that varies from tissue to tissue. A 40:1 ratio is thought to be physiological for most tissues [118]. Insulin triggers the NAD–NADH-dependent enzyme epimerase to function in accordance with tissue needs. When there is insulin resistance in the systemic milieu, this stimulus is lost. The main inositol isomers are distributed differently in the three insulin target organs—adipose tissue, liver, and skeletal muscle—and each requires a substantially higher proportion of DCI to maintain homeostasis. In the presence of epimerase deficiency, less MI can be converted into DCI, resulting in a state of relative DCI deficiency and the promotion of insulin resistance [118]. This, in turn, causes the metabolic complications of hyperinsulinemia, a feature of PCOS.

In PCOS-afflicted women who are otherwise infertile, MI intake has been demonstrated to improve ovulation and responsiveness to fertility treatments. A recent systematic analysis that evaluated the effects of MI in PCOS-affected women concluded that MI supplements improved PCOS-related hormonal and reproductive issues. Additionally, it improved oocyte maturation as well as follicular development and raised the likelihood of clinical pregnancies in PCOS patients. With MI therapy, ovulation induction time and recombinant FSH dosage requirements were both dramatically reduced [119]. As a result, it positively modifies the reproductive axis. Treatment with MI markedly decreased LH, prolactin, androstenedione, insulin, and LH/FSH ratio concentrations, in addition to enhancing insulin sensitivity [120]. Since MI supplements are often well-tolerated at the current dose recommendations of 2–4 g/day with few safety concerns, their clinical use in the management of PCOS is worth taking into account. It has been demonstrated that DCI plays a role in insulin metabolism. Both at baseline and after the administration of glucose loads, women with PCOS are reported to have decreased serum levels of DCI. In PCOS patients, DCI therapy has been shown to improve endocrine, metabolic, and reproductive parameters by lowering blood pressure, lipid, and insulin levels as well as improving the maturity and quality of oocytes significantly, while reducing oxidative stress in follicular fluid [121,122,123,124]. All PCOS symptoms, signs, and test abnormalities may be improved with Myo- + D-chiro-inositol (MDI) therapy. Together, the two inositols should be able to increase the necessary inositol concentrations in the ovary and systemic circulation, resolving the ovarian inositol paradox. The metabolic characteristics of PCOS will be treated by MI, which will correct systemic insulin resistance. A healthy intraovarian environment will be produced simultaneously by sufficient DCI levels, which will treat hyperandrogenism, enhance menstrual regularity, and promote ovulation as well as fertility [120]. Exogenous DCI administration may be a method of circumventing defective epimerase activity and achieving the downstream metabolic effects of insulin in DCI-deficient tissue. Due to the unidirectional nature of epimerase activity, administering DCI by itself will not be able to imitate the effects of MI. As a result, it makes sense to provide a combination of both to ensure optimal insulin sensitivity. At the same time, the beneficial effect of MI on ovarian physiology could be attributed to its low conversion into DCI. Lower doses of MI may be enough if coadministered with DCI, according to this hypothesis. Considering the physiologic and pharmacological evidence presented above, we propose the pragmatic use of inositol therapy in the prevention and management of PCOS. Through the use of MDI therapy, MI and DCI deficiency can be treated concurrently, which may help to reduce the menstrual/ovulatory, metabolic, and cutaneous hyperandrogenic symptoms of PCOS. The above being the case, MDI, both as a monotherapy and in combination, is a sensible treatment option for PCOS management. Ongoing research will contribute to increased confidence in the scientific application of these molecules.

### 6.4. MicroRNA (miRNA) Therapy

Evidence is growing about the therapeutic potential of miRNAs in the management of numerous diseases, including PCOS [125,126]. The post-transcriptional regulation of gene expression is carried out by miRNAs, which are small non-coding RNAs with a length of about 22 nucleotides. They attach selectively to the 3’ untranslated region (UTR) of the target genes, inhibiting their translation and/or causing instability [127]. Their binding to target messenger RNA (mRNA) causes mRNA cleavage, translational repression, and mRNA decay [128,129]. These small RNA molecules participate in a wide range of physiological activities, including differentiation, apoptosis, proliferation, inflammation, metabolism, and stress responses [125]. Nearly 30% of human genes are known to act as miRNA target sites [125]. A single miRNA can influence the activity and expression of a wide range of target genes, and the amplification or inhibition of a miRNA signal via the regulatory feedback mechanism may result in a significant alteration in miRNA expression that contributes to various diseases, including ovarian cancer, endometriosis, cardiovascular disease, inadequate ovarian responses, diabetes, PCOS, and others [127,130,131]. In recent years, mounting data have suggested that aberrant miRNA expression is seen in the theca cells, adipose tissue [132], follicular fluid, cumulus cells, granulosa cells [133,134], serum [135], and peripheral blood leukocytes of PCOS-affected women in addition to playing a critical role in the onset and progression of the condition [125]. It regulates steroid hormone synthesis, follicular development and maturation, adipogenesis, and insulin signaling pathways. Recent research has found differences in the expression of certain miRNAs between women with PCOS and healthy women, suggesting that miRNAs may be crucial in the emergence and progression of PCOS [135]. In PCOS, miRNAs contribute to inflammation, ovarian insulin sensitivity, hyperinsulinemia, and oocyte quality [125]. MiRNAs can be used to distinguish between various phenotypes according to the Rotterdam criteria and may develop into distinct and reliable biomarkers for the diagnosis of PCOS due to their specificity.

Certain miRNAs might serve as novel biomarkers for a PCOS-related aberrant metabolism, decreased oocyte quality, and low endometrial receptivity [126]. For instance, by controlling steroidogenesis, inducing proliferation, and apoptosis, miR-182 and miR-15a are crucial for the physiology of GCs in the ovaries; however, their levels were noticeably low in the ovarian cells of a PCOS rat model [136]. Therefore, by targeting the GnRH pathway, the expression of these miRNAs may affect the timing of the development as well as the maturation of the oocyte and subsequently folliculogenesis, making it a potential target for assessing ovulation in PCOS. Similarly, several miRNAs regulating steroidogenesis are differentially expressed in the FF of women with PCOS and normal control women [126]. An in-depth understanding of the correlation between miRNAs and the synthesis of steroid hormones will potentially aid in the diagnosis of PCOS and help predict its metabolic consequences. Additionally, via influencing the expression of GLUT4, proteins, and enzymes involved in glucose metabolism, miRNA expression regulates glucose metabolism, the insulin signaling system, and the development of IR in women with PCOS. It is generally known that miRNAs have a significant impact on cholesterol homeostasis and lipid metabolism. MiRNAs linked with LDL-C metabolism, BMI, and adipogenesis, such as miR-128-1, miR-185, miR-148a, and miR-375, exhibit abnormal expressions in PCOS. A strong correlation between miRNAs, obesity, and dyslipidemia establishes its potential as a therapeutic target in the treatment of the metabolic symptoms of PCOS. Considering all of this, miRNAs could potentially be clinical biomarkers for the diagnosis of PCOS and a therapeutic target for the treatment of PCOS. Potential miRNA-based therapeutic strategies for PCOS include the restoration or inhibition of miRNA function through miRNA mimics and inhibitors (anti-miRs). An altered miRNA profile holds the potential for new methods with which to stratify PCOS patients and may contribute to as well as partially explain heterogeneity in PCOS women. This may facilitate a personalized level of medical care for women with PCOS and predict outcomes. High-throughput miRNA profiling and sequencing can be used as a general tool with which to examine various signaling pathways, enhance clinical management, and choose an appropriate infertility treatment based on aberrant follicular development in PCOS patients. MiRNA can provide us with the exquisite analysis, prevention, and management of reproductive disorders, such as PCOS, with the use of cutting-edge technology and searchable databases. The primary goal is to carry out extensive replication experiments to find specific miRNAs that have significant modulating effects on PCOS. Considering the fact that miRNAs often exist in families, an increasingly crucial approach is to regulate the levels of entire miRNA families or coregulated miRNAs together as well as separately to define their roles in intact tissues or organs. Additionally, we may examine the functional significance of individual miRNAs in the pathology of PCOS and confirm their functionality both in vivo and in vitro. The potential miRNA-based therapeutic options will provide new horizons and compelling alternatives for the treatment of PCOS and its related metabolic complications. Small interfering RNA (siRNA) therapies, anti-miRNA oligonucleotides, and miRNA mimics are all currently being studied. There is not currently a medication that specifically targets the miRNAs involved in PCOS; however, researching related miRNAs may open up new avenues for future PCOS research that uses miRNAs as biomarkers. Several clinical trials are currently underway to evaluate the potential therapeutic effects of targeting miRNAs in obesity and its associated metabolic disorders, which may also benefit women with PCOS.

### 6.5. Interleukin (IL)-22 Therapy

IL-22, a cytokine produced by intestinal immune cells, is crucial in the regulation and function of host defense as well as inflammatory diseases. IL-22 promotes wound healing and restores tissue integrity in addition to homeostasis in tissues and organs with high IL-22 receptor expression by preventing cell death and tissue damage brought on by inflammation and infection. IL-22 exerts an important role in eliciting antimicrobial immunity and maintaining mucosal barrier integrity within the intestine. It shows a variety of metabolic benefits, as it improves insulin sensitivity, preserves the gut mucosal barrier and endocrine functions, decreases endotoxemia and chronic inflammation, and regulates lipid metabolism in the liver and adipose tissues [24]. A recent study reported reduced levels of IL-22 in the serum and follicular fluid of PCOS patients, in addition to the fact that IL-22 administration could help to improve insulin resistance, ovarian dysfunction, and infertility in intestinal bacteria or a prenatal AMH-induced PCOS mice model [137]. In high-androgen-induced PCOS mice, Xinyu et al. found that IL-22 could reverse insulin resistance, disrupt the estrous cycle as well as aberrant ovarian morphology, and decrease the embryo number. This study showed that insulin sensitivity and ovarian functions in PCOS were modulated by IL-22-associated browning of white adipose tissue, indicating that IL-22 may help treat PCOS with a hyperandrogenism phenotype [138]. Clinical trials have shown that the administration of exogenous IL-22 could provide therapeutic benefits [139]. The above being the case, the IL-22 pathway may act as a novel target for therapeutic intervention in metabolic disorders such as PCOS.

### 6.6. Restoration of the Gut Microbiome: Towards Treatment

**Figure 3:**
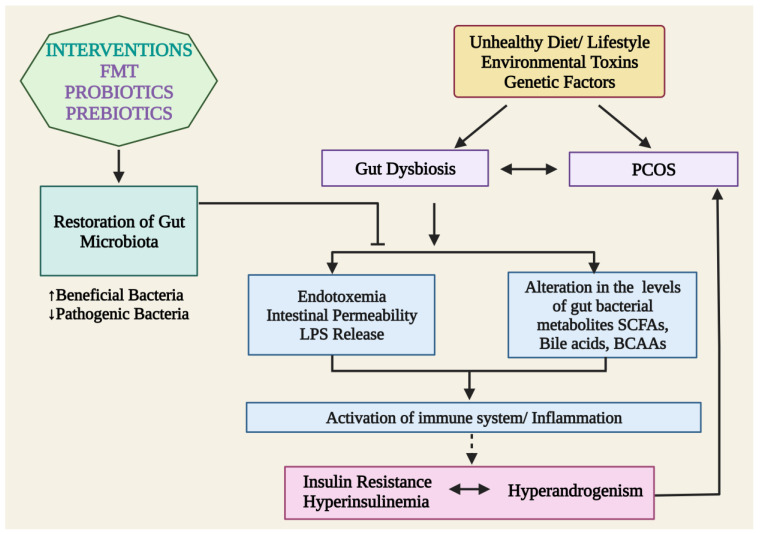
Plausible mechanisms of action of probiotics, prebiotics, and FMTs in restoring gut microbiota and alleviating PCOS. LPS: lipopolysaccharides; SCFAs: short-chain fatty acids; and BCAAs: branched-chain amino acid and FMTs: Fecal microbiota transplantation.

Dysbiosis of gut microbiota may be a potential driver in the development of PCOS symptoms. With a greater understanding of the microbiome’s role in the pathophysiology of PCOS, significant attempts have been undertaken to create novel management strategies for this disorder. Probiotics (living microorganisms), prebiotics (sources of food for beneficial gut bacteria), synbiotics, and more recent therapies including FMTs are among the treatment possibilities for the altered gut microbiome that causes PCOS [140] (Figure 3).

#### 6.6.1. Probiotics and Prebiotics

According to the World Health Organization (WHO), probiotics are “live microorganisms that, when administered in adequate amounts, confer a health benefit on the host” [141]. Probiotic microorganisms are naturally found in fermented foods and have anti-oxygenic, anti-microbial, and anti-inflammatory properties, as well as the ability to improve metabolic parameters, modulate intestinal microbiota, and regulate the immune system. The most popular bacterial genera utilized as probiotics include *Lactobacillus, Bacillus, Bifidobacterium, Streptococcus*, and *Enterococcus* [142]. Probiotic supplements have been demonstrated to improve the metabolic profile of PCOS [143,144,145,146]. According to Ahmadi et al., probiotic supplementation (*L. acidophilus, L. casei,* and *B. bifidum*) for 12 weeks led to a statistically significant reduction in weight and BMI in PCOS patients compared to the placebo, with positive effects on glycemia, TGs, and VLDL-C [143]. Women with PCOS reported similar outcomes after taking supplements *of L. casei, L. acidophilus, L. rhamnosus, L. bulgaricus, B. breve, B. longum,* and *Streptococcus thermophiles* for 8 weeks. These supplements led to a significant drop in plasma glucose and serum insulin levels [145]. Recent meta-analysis reports indicated that probiotics have a significant impact on the regulation of hormonal and inflammatory indicators, with a significant decrease in the free androgen index (FAI) and malondialdehyde (MDA), an increase in SHBG and nitric oxide (NO), and an improvement in the weight, BMI, insulin, HOMA-IR, TGs, VLDL-cholesterol, hirsutism, and total testosterone of PCOS patients [147,148,149].

Prebiotics are fermented substances that cause specific changes in the composition and/or activity of a host’s gut microbiota. Prebiotics include polyunsaturated fatty acids (PUFAs), polyphenols, and carbohydrates, such as inulin, lactulose, fructans, fructooligosaccharides (FOS), galactooligosaccharides (GOS), and xylooligosaccharides (XOS). Prebiotics work through a variety of mechanisms, including the selective induction of beneficial intestinal bacteria growth or activity, fermentation via gut microbiota, and preventing pathogen colonization by interacting with them. Prebiotics stimulate the growth of both Bifidobacterium and Lactobacillus, which results in a significant decrease in fasting plasma glucose, the levels of serum TG, total cholesterol, and LDL-C, as well as a significant increase in HDL-C levels, according to some studies [144,145]. These results indicate that prebiotics has positive effects on metabolic markers and immunomodulatory properties [150]. According to a study, the regular consumption of resistant dextrin, which is prebiotic, can help in the regulation of metabolic parameters and could reduce hyperandrogenism as well as menstrual cycle abnormalities in PCOS women [151]. Additional studies are required to elucidate the effectiveness of different prebiotics and probiotic strains and dosages, to determine the ideal course of treatment, and to show the positive effects of probiotics, prebiotics, and synbiotics on clinical outcomes in PCOS.

#### 6.6.2. Fecal Microbiota Transplantations (FMTs)

FMTs are an intriguing and innovative biotherapeutic approach that involve transplanting the fecal fluid of treated healthy people into the intestines of diseased patients to treat certain diseases by reconstructing the intestinal flora. FMTs have now become a successful method for treating metabolic disorders. By boosting the production of SCFAs, especially butyrate, which protects the integrity of the epithelial barrier, FMTs can reduce intestinal permeability. FMTs activate the intestinal adaptive immune response through the TLR pathway, which speeds up the production of immunoglobulins and defends the intestinal mucosa [152]. FMTs can regulate the diversity of gut microbiota, blood sugar level, and insulin sensitivity, in addition to mediating the release of inflammatory cytokines [153]. It might be a viable cutting-edge PCOS treatment approach. FMTs reduce blood androgen levels, elevate estrogen levels, and help to maintain a regular menstrual cycle, according to an in vivo study [154]. In PCOS rat models, treatment with Lactobacillus and FMTs improved hyperandrogenism and influenced insulin function [154]. In a study, PCOS rat models treated with FMTs had improved menstrual cycles and decreased androgen biosynthesis as compared to the untreated group [155]. Based on numerous mechanisms and research, a new theory is being posited that predicts that the combination of FMTs and curcumin will be a successful and long-lasting treatment for PCOS with substantially lower rates of remission [156]; however, aside from murine models, there are no clinical reports on the use of FMT to treat PCOS. Prospective results from laboratory studies should motivate additional human investigations.

## 7. Future Perspectives

Clinically, PCOS is a complex condition with lifelong complications, and it is becoming a prevalent disorder in women of reproductive age. The most challenging aspects of this syndrome are the imprecise diagnostic criteria and the enormous complexity of its characteristics. The timely implementation of personalized therapy approaches will enhance PCOS management overall, reduce comorbidities, and improve quality of life. To improve its prognosis, early detection and treatment are necessary for females who may experience infertility during their reproductive years. Key gene polymorphisms might be useful for early PCOS subtype diagnosis and screening. Further studies on the genetics and pathophysiology of PCOS will be necessary for identifying both effective prevention strategies and therapeutic approaches. Additional research is required to check if the composition of intestinal microbes changes as a result of steroid alterations and the processes behind it in PCOS patients. Prebiotic, probiotic, and synbiotic supplementation in women with PCOS appears to improve numerous biochemical findings and have favorable effects, although the mechanisms are yet unknown. More research is needed to determine the significance of these medications in PCOS therapy or, maybe, prevention. Randomized clinical trials are needed to elucidate the mechanisms underlying the association between gut microbiota dysbiosis and PCOS. Future comprehensive and functional research will allow for the use of gut microbiota as a biomarker for PCOS, and the targeted personalized manipulation of gut microbiota will help advance the research. There is no panacea because treatments have, so far, been directed at symptoms rather than the illness itself. Extensive efforts should be undertaken to thoroughly investigate the syndrome to improve therapy and delay the serious long-term impacts of the disease on patients’ health. Several emerging therapies for the treatment of T2DM may have direct utility in the management of the metabolic aspects of PCOS; however, clinical studies are needed to evaluate their clinical efficacy and safety in women with PCOS. Additional investigations are required to prove the potential of emerging therapeutics, such as miRNA therapy, IL-22 therapy, and others, in positively treating PCOS.

## Figures and Tables

**Table 1:** Four major clinical phenotypes of PCOS.

Feature	Phenotype A	Phenotype B	Phenotype C	Phenotype D
Biochemical/clinical hyperandrogenism	+	+	+	−
Chronic anovulation	+	+	−	+
Polycystic ovaries	+	−	+	+

## Data Availability

Not applicable.
